# Supplementary material for: High-speed blind structured illumination microscopy via unsupervised algorithm unrolling
Source: Nat Commun. 2026 Jan 23;17:1967. doi: 10.1038/s41467-026-68693-w (PMC12932644; doi:10.1038/s41467-026-68693-w)
Supplement: Supplementary file 1 — Supplementary Information [file 41467_2026_68693_MOESM1_ESM.pdf]

Supplementary information for:

High-speed blind structured illumination microscopy via unsupervised algorithm unrolling

Zachary Burns[1, ‡], Junxiang Zhao[1, ‡], Ayse Z. Sahan[2,3], Jin Zhang[2,4,5], Zhaowei Liu[1,6,7,*]

[1] *Department of Electrical and Computer Engineering, University of California, San Diego, 9500 Gilman Drive, La Jolla, California 92093, USA*

[2] *Department of Pharmacology, University of California, San Diego, 9500 Gilman Drive, La Jolla, California 92093, USA*

[3] *Biomedical Sciences Graduate Program, University of California, San Diego, 9500 Gilman Drive, La Jolla, California 92093, USA*

[4] *Department of Bioengineering, University of California, San Diego, 9500 Gilman Drive, La Jolla, California 92093, USA*

[5] *Department of Chemistry & Biochemistry, University of California, San Diego, University of California, San Diego, 9500 Gilman Drive, La Jolla, California 92093, USA*

[6] *Material Science and Engineering Program, University of California, San Diego, 9500 Gilman Drive, La Jolla, California 92093, USA*

[7] *Center for Memory and Recording Research, University of California, San Diego, 9500 Gilman Drive, La Jolla, California 92093, USA*

‡ These authors contributed equally to this work

*Corresponding author email address: zhaowei@ucsd.edu

Contents

S1. Comparison with current blind-SIM literature

S2. Forward model for blind-SIM

S3. Error analysis of illumination uniformity estimator

S4. Gradient Descent, Nesterov, and Conjugate Gradient algorithm details

S5. Architecture for unrolled network CNN

S6. UBSIM training details

S7. Generation of simulated training datasets

S8. Comparison of overfitting to noise

S9. Illumination pattern reconstruction

S10: Evaluation of iteration block number on performance

S11. Imaging system experimental setup

S1: Comparison with current blind-SIM literature

Title	Method	Live Cell data?	Video data?	Limitations
Structured illumination microscopy using unknown speckle patterns[1] (2012)	Iterative physics-based model	No (fixed tissue only)	No	• Slow reconstruction even with conjugate gradient acceleration<br>• No dynamic, or live cell imaging demonstrated
Structured illumination fluorescence microscopy with distorted excitations using a filtered blind-SIM algorithm[2] (2013)	Iterative physics-based model	No (fixed cell only)	No	• Slow iterative reconstruction method<br>• No live cell or video data
Fluorescent microscopy beyond diffraction limits using speckle illumination and joint support recovery[3] (2013)	Iterative physics-based model	No (fixed cell only)	No	• Slow iterative reconstruction method<br>• No live cell or video data
Structured illumination microscopy with unknown patterns and a statistical prior[4] (2017)	Iterative physics-based model	No	No	• Slow iterative reconstruction method<br>• No video or live cell demonstrations
Joint Reconstruction Strategy for Structured Illumination Microscopy With Unknown Illuminations[5] (2017)	Iterative physics-based model	No	No	• Requires regularization parameter tuning<br>• Accelerated iterative method still requires more iterations than UBSIM<br>• No video or live cell demonstrations
On the Superresolution Capacity of Imagers Using Unknown Speckle Illuminations[6] (2018)	Iterative physics-based model	No	No	• Purely theoretical paper<br>• Slow iterative reconstruction method<br>• No video or live cell demonstrations
Super-resolved live-cell imaging using random illumination microscopy[7] (2021)	Iterative physics-based model	Yes	Yes	• Slow iterative reconstruction

Deep learning for blind structured illumination microscopy[8] (2022)	Deep learning	Yes	No	• Limited experimental evaluation<br>• No rigorous exploration of generalizability<br>• Requires supervised training scheme
Fast super-resolved reconstructions in fluorescence random illumination microscopy (RIM)[9] (2024)	Iterative physics-based model	No	No	• Accelerated iterative method<br>• Resolution improvement cannot reach 2x in non-noiseless demonstrations<br>• Limited quantitative resolution evaluation<br>• No experimental live cell data

Supplementary Table 1: Summary of current blind-SIM papers and their limitations compared to this work.

S2: Forward model for blind-SIM

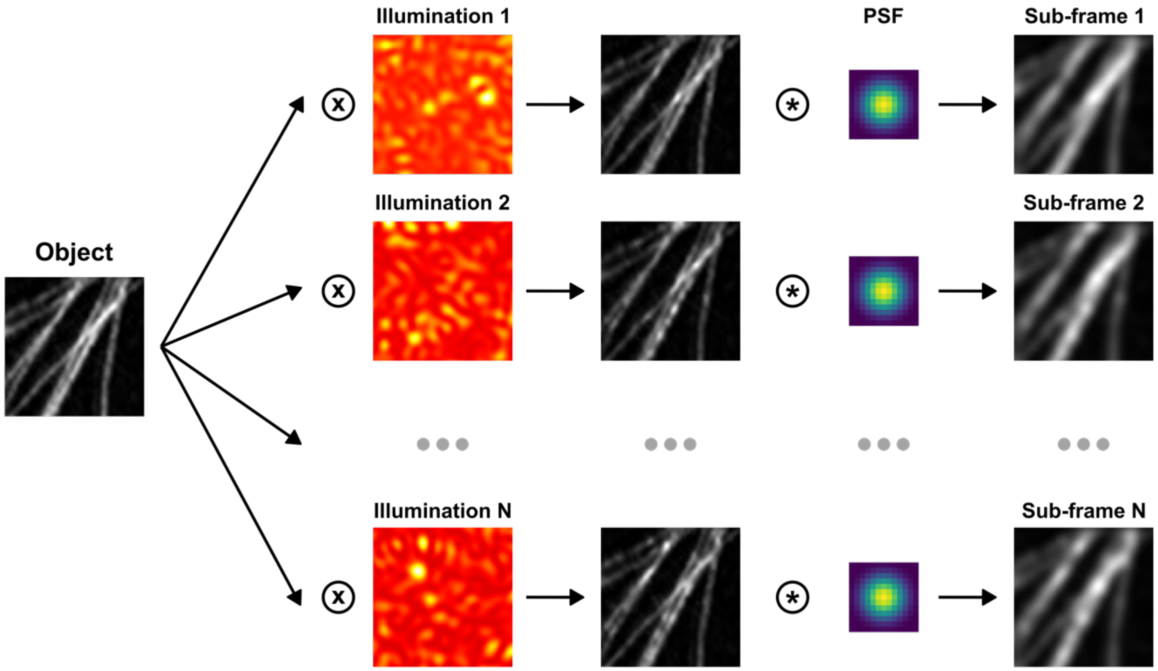

Supplementary Figure 1: Forward model for blind structured illumination microscopy. A fluorescently labeled object is illuminated with a series of N speckle patterns which modulates the emitted fluorophore intensity. The modulated object is then convolved with the detection objective point spread function (PSF) to produce a sub-frame[1].

S3: Error analysis of illumination uniformity estimator

In this section, an error analysis for the uniformity assumption of the blind-SIM algorithm is presented. The bias, variance, and root mean square error of the estimator is derived as a function of the number of sub-frames used. We also discuss how error in the uniformity assumption affects the reconstructed image.

The uniformity assumption is defined as:

$$\sum_{l=1}^{L} I_l \approx L I_0 \tag{1}$$

The assumption is that the sum of all illumination patterns equals the average intensity multiplied by the number of frames.

The fractional error is then defined as:

$$E_l = \frac{\sum_{l=1}^{L} I_l - L I_0}{L I_0} \tag{2}$$

For fully developed speckle[10], the intensity follows an independent and identically distributed (i.i.d) exponential random variable with mean I_0 and variance I_0^2.

The expected value of the error (bias of the estimator) is then:

$$\mathbb{E}(E_l) = \frac{\mathbb{E}(\sum_{l=1}^{L} I_l) - L I_0}{L I_0} = \frac{\sum_{l=1}^{L} \mathbb{E}(I_l) - L I_0}{L I_0} = \frac{L I_0 - L I_0}{L I_0} = 0 \tag{3}$$

The variance of the estimator is:

$$Var(E_l) = Var\left(\frac{\sum_{l=1}^{L} I_l - L I_0}{L I_0}\right) = Var\left(\frac{\sum_{l=1}^{L} I_l}{L I_0} - 1\right) = \frac{Var(\sum_{l=1}^{L} I_l)}{(L I_0)^2} = \frac{L I_0^2}{L^2 I_0^2} = \frac{1}{L} \tag{4}$$

The mean squared error can then be calculated from the decomposition:

$$MSE(E_l) = Bias(E_l)^2 + Var(E_l) = \frac{1}{L} \tag{5}$$

Giving a root mean squared error of:

$$RMSE(E_l) = \frac{1}{\sqrt{L}} \tag{6}$$

Therefore, the residual error in the uniformity constraint will decrease with an inverse square relationship with the number of sub-frames used for reconstruction. Adding more frames will always result in reduced error, however at a diminishing rate of return.

The presence of error in the uniformity constraint will lead to (1) a multiplicative shading of the resulting image and (2) higher noise which will result in granular artifacts[1,4]. The resolution of reconstructed image is ultimately determined by the spatial frequency support of the optical transfer function (OTF) and the illumination speckle. However, artifacts from shading and lowered signal to noise ratio from fewer sub-frames may reduce apparent resolution. Ultimately, the number of sub-frames used is a parameter to be chosen by the user that will cause a tradeoff between shading accuracy and temporal resolution.

S4. Gradient Descent, Nesterov, and Conjugate Gradient algorithm details

Gradient descent:

For blind-SIM[1], the iterative update equations for ξ and i (the auxiliary variables for the object and illumination patterns used to enforce positivity) are:

$$\begin{aligned} \xi_n &= \xi_{n-1} + \alpha_n d_{n,\xi} \\ i_{l,n} &= i_{n-1} + \alpha_n d_{l,n,i} \end{aligned} \quad (7)$$

Where α is the step size, d is the updating direction and n is the iteration number.

For standard gradient descent, the updating directions are simply the gradients g of the cost function with regard to each variable and the updates equations are:

$$\begin{aligned} \xi_n &= \xi_{n-1} + \alpha_n g_{n,\xi} \\ i_{l,n} &= i_{l,n-1} + \alpha_n g_{l,n,i} \end{aligned} \quad (8)$$

At each iteration, a backtracking line search is performed to find α_n so that there is a sufficient decrease in the cost function (Armijo condition, see Algorithm 3.1 of *Numerical Optimization*, Springer (2006))[11].

Nesterov's accelerated method:

Nesterov accelerated gradient[12] descent builds upon regular gradient descent by adding a momentum term and computing the update gradient at a look-ahead point.

For blind-SIM, the look-ahead points are then:

$$\begin{aligned} \omega_n &= \xi_n + \beta_n(\xi_n - \xi_{n-1}) \\ \zeta_{l,n} &= i_{l,n} + \beta_n\left(i_{l,n} - i_{l,n-1}\right) \end{aligned} \quad (9)$$

Where β_n is the momentum term. The update equations are:

$$\begin{aligned} \xi_{n+1} &= \omega_n + \alpha_n \nabla f(\omega_n) \\ i_{l,n+1} &= \zeta_{l,n} + \alpha_n \nabla f\left(\zeta_{l,n}\right) \end{aligned} \quad (10)$$

Where ∇f is the gradient of the cost function evaluated at the look-ahead points.

Similar to gradient descent, the step size α_n is chosen at each step to satisfy the sufficient decrease condition via backtracking line search.

Conjugate gradient method:

For conjugate gradient descent[13], previous update directions are considered to ensure that the successive steps are conjugate and incorporate curvature information without the use of the Hessian. The update directions are:

$$
\begin{aligned}
d_{n,\xi} &= g_{n,\xi} + \gamma_{n,\xi} d_{n-1,\xi} \\
d_{n,l,i} &= g_{n,l,i} + \gamma_{n,l,i} d_{n-1,l,i}
\end{aligned}
\tag{11}
$$

Where the γ are defined as:

$$
\gamma_{n,\xi} = \frac{\langle g_{n,\xi} \mid g_{n,\xi} - g_{n-1,\xi} \rangle}{\left\| g_{n-1,\xi} \right\|^2} \tag{12}
$$

$$
\gamma_{n,l,i} = \frac{\langle g_{n,l,i} \mid g_{n,l,i} - g_{n-1,l,i} \rangle}{\left\| g_{n-1,l,i} \right\|^2} \tag{13}
$$

At each iteration, the step size α is determined by an inexact line search to satisfy the strong Wolfe conditions of (1) sufficient decrease and (2) curvature of descent direction (see Algorithm 3.5 and 3.6 of *Numerical Optimization*, Springer (2006))[11].

Parameter Note:

For the iterative reconstruction methods used in this work we use an initial step size of 0.1 and an Armijo constant (c1) of 1e-4. For the conjugate gradient method, a Wolfe parameter value (c2) of 0.1 is used. For the Nesterov method a momentum value of 0.9 is used. For each backtrack, the step size is multiplied by a factor of 0.5.

S5: Architecture for unrolled network CNN

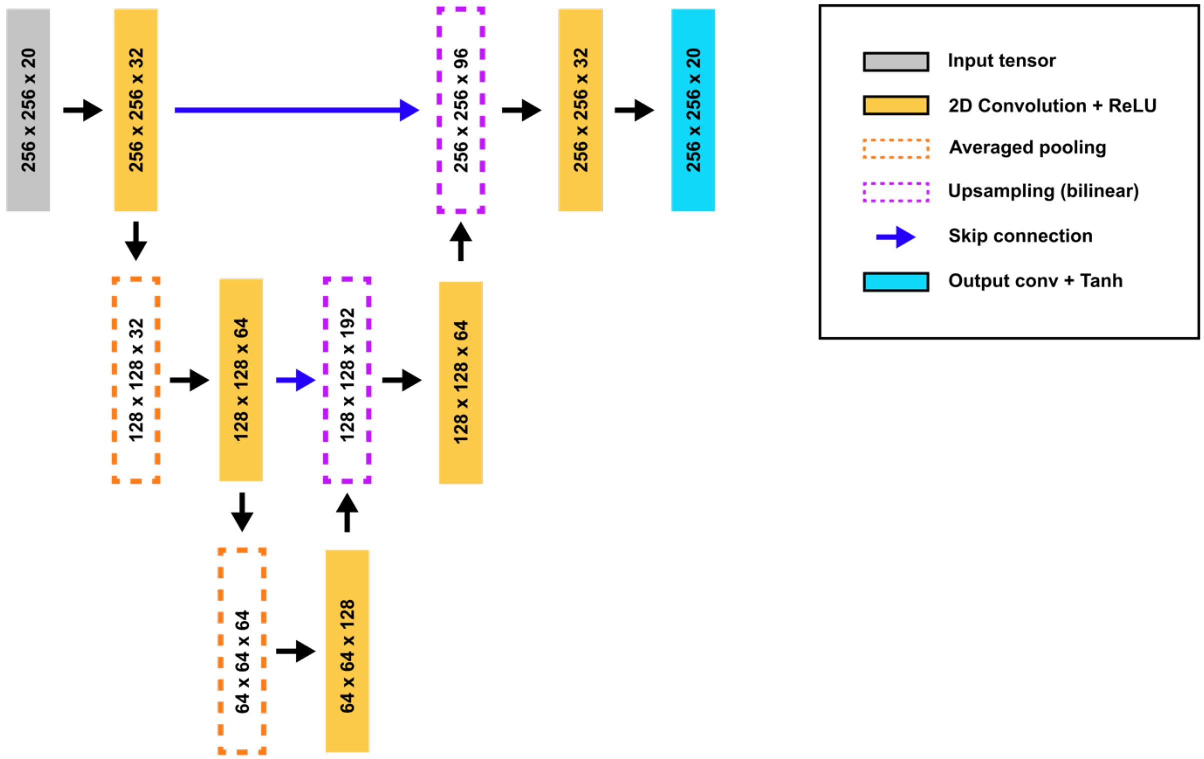

Supplementary Figure 2: Architecture for unrolled network CNN. The neural network used in UBSIM is a modified U-Net architecture with 2 skip connections[14]. Average pooling is used to downsample the feature maps and bilinear interpolation is used to upsample. The output convolution has a hyperbolic tangent activation function to limit the output range.

S6: UBSIM training details

UBSIM models for this work were written in PyTorch using version 2.1.2 with Python version 3.10.14. Models are trained using simulated datasets generated by the process described in supplementary section 7. Models are trained on a Nvidia A6000 Ada GPU. For a typical training run, the hyperparameters are as follows. The initial learning rate is 1e-4 and reduces by a factor of 0.5 upon a plateau of 5 epochs. Models were trained until the improvement in validation loss became negligible and image quality metrics plateaued. For mixed object datasets we trained for 200 epochs and for single object datasets we trained for 50 epochs. The Adam optimizer was used, and batch size was 1. During training, a normalized version of the blind-SIM cost function was used as the UBSIM loss function. The normalized cost function takes the squared residual and divides by the average intensity image across the sub-frames. This prevents denser objects from having a larger effect on the network optimization over sparser objects. Models used 1,200 images for training, 100 images for validation, and 100 images were reserved for the test set. Two models were trained with PSFs with full width half maximum values of 5 and 6 pixels respectively. We found that these models could generalize well during inference to cases where the PSF width varies modestly from the training PSF width.

S7: Generation of simulated training datasets

All models in this paper are trained on simulated training data. In the main text, Figures 2-4 are evaluated on simulated data that was not included in the training process (test set). Figures 5-6 are evaluated on experimental data. The dataset and the MATLAB code to generate it are provided on Zenodo (https://zenodo.org/records/17852915). The simulated dataset is derived from experimentally captured cell data in the publicly available BioSR dataset[15]. The data generation process is described as follows. First, a random cropped area is selected from between the 4 labeled organelle types in the BioSR dataset (actin, endoplasmic reticulum, microtubules, and clathrin-coated pits). This cropped area serves as the ground truth image. The cropped images are then rescaled so that feature sizes can be seen with a two-fold resolution improvement and undergo background subtraction. Next, the sub-frames for each image are created by generating a speckle pattern, multiplying the ground truth image with the speckle pattern, and convolving the product with a point spread function (PSF). The generated speckle patterns are made such that the spatial frequency has the same cutoff as the PSF. This is meant to simulate a system where the illumination and detection objective have the same numerical aperture. Additionally, the speckle contrast is randomly varied within the training dataset. The raw sub-frames are then downsampled by a factor of two and additive white Gaussian noise is added. The overall stack of sub-frames is normalized to the range [0,1]. For generalization testing (Fig. 4) separate datasets were created that included only objects of one type (ER, CCP, or MNIST) but otherwise followed the same generation process described here.

S8. Comparison of overfitting to noise

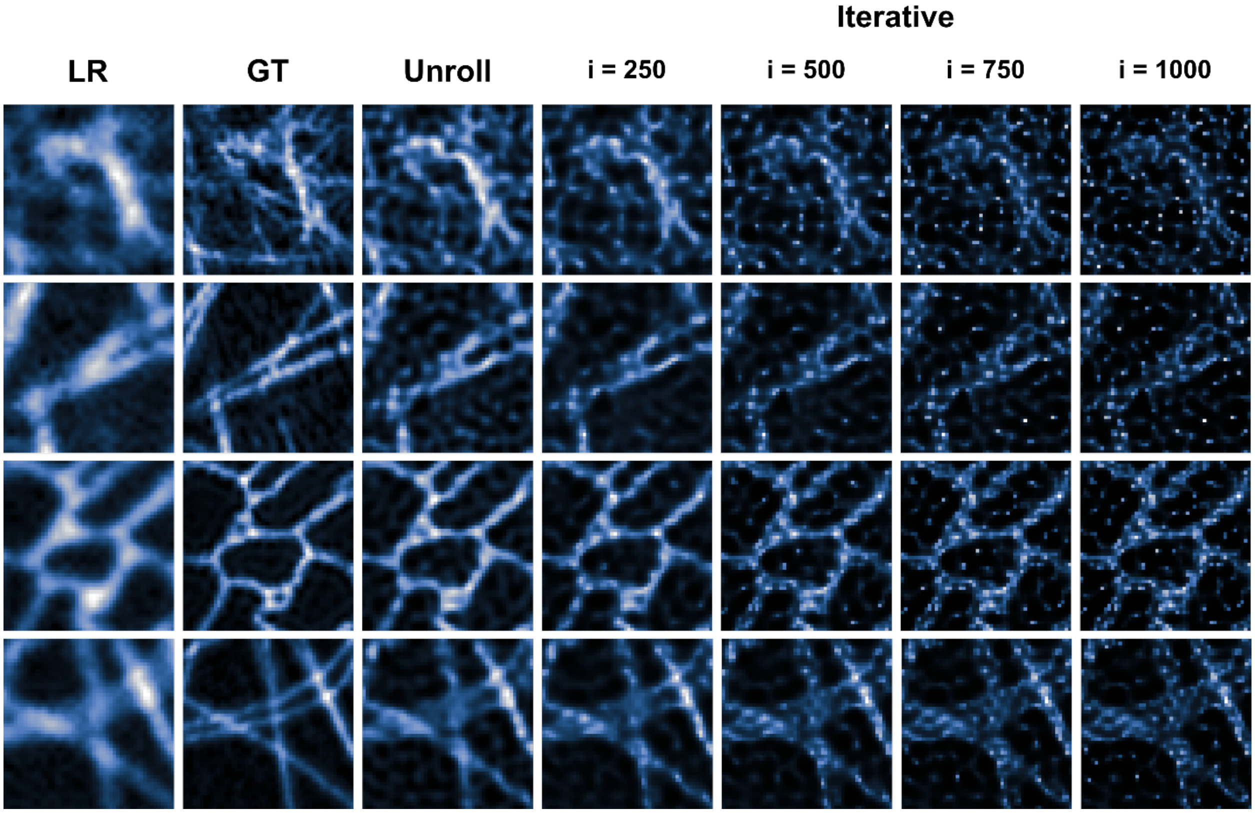

Supplementary Figure 3: Comparison of noise overfitting between iterative and unrolled blind-SIM. Examples of different images reconstructed with unrolled and iterative blind-SIM with low signal-to-noise ratios. Columns from left to right: low-resolution image, ground-truth image, UBSIM reconstruction, iterative blind-SIM reconstructions with varying number of iterations. i is the number of iterations.

As noted in the original blind-SIM paper by Mudry and colleagues[1], "both blind-SIM and deconvolution algorithms are not well regularized. They converge towards meaningless images in which the noise is amplified." This means that for iterative blind-SIM there is an uncertain stopping criterion. Increasing the number of iterations past a certain point may lower the cost function, however the reconstructed image quality will become worse as the data-fit term in the cost function is minimized by overfitting to noise. Therefore, a challenge of using iterative blind-SIM is determining when to stop the iteration process.

We examined the reconstruction results of UBSIM and iterative blind-SIM when there is a low signal-to-noise ratio. As can be seen in Supplementary Figure 3, as the number of iterations increases for iterative blind-SIM, the results converge to sparse, dot-like images where the noise is amplified. UBSIM, on the other hand, produces results that are fairly well regularized. While there are some artifacts from noise, the overall object structure is still dominant. Therefore,

another benefit of UBSIM over iterative-blind SIM is that it removes the uncertain stopping criterion problem.

We note here that the simulated data are corrupted with additive white Gaussian noise with a SNR of 2 and that the UBSIM model was trained on a training dataset with a SNR of 16. Thus, UBSIM generalizes well to cases where the noise level is higher than the training dataset.

S9. Illumination pattern reconstruction

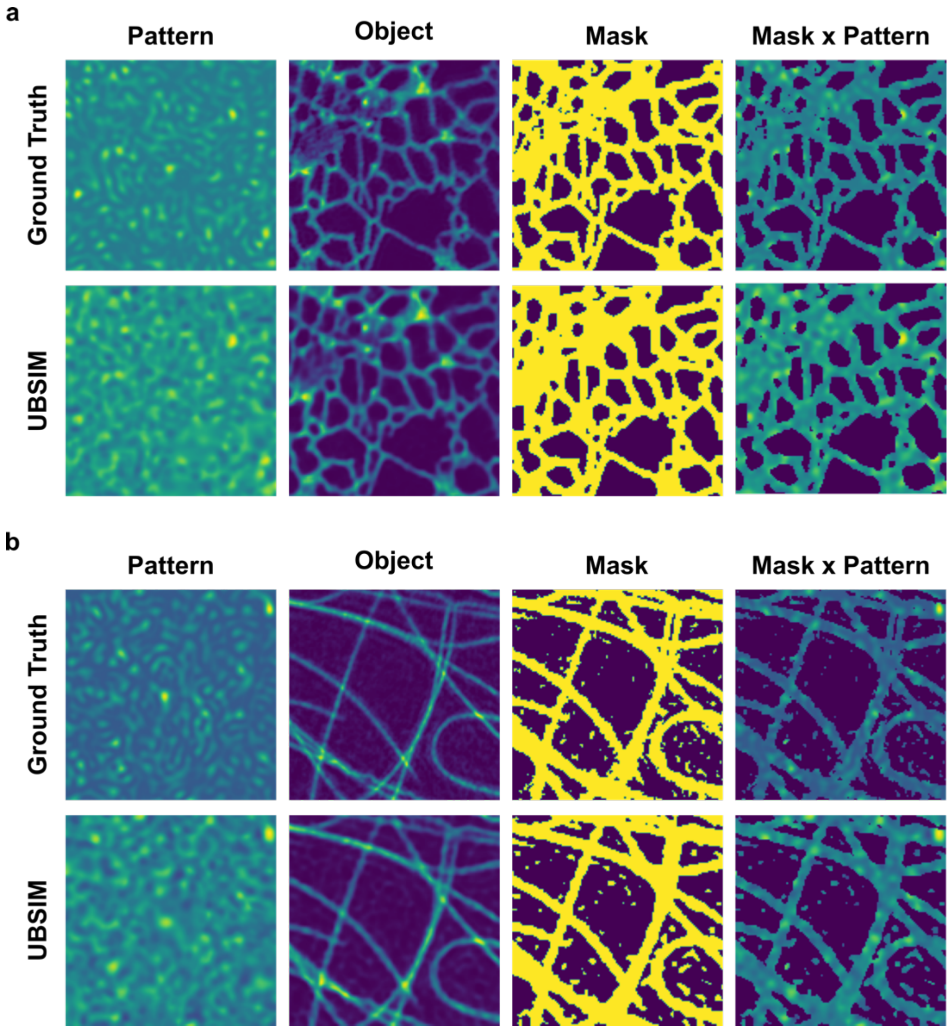

Supplementary Figure 4: Evaluation of UBSIM illumination pattern reconstruction. Two examples of UBSIM reconstruction of illumination patterns for (a) endoplasmic reticulum and (b) microtubules. The ground-truth and reconstructed illumination patterns and objects are presented along with a mask indicating where the object is present in the frame. The last column uses the mask to indicate the regions of the illumination patterns where the object is present.

Blind-SIM solves for both the object and the illumination patterns for each sub-frame. In the manuscript we focus on the super-resolution objects reconstructed by UBSIM. Here we qualitatively examine some examples of UBSIM reconstruction of illumination patterns from the same simulated dataset used in Fig. 2 in the main text.

It is important to note one point: blind-SIM can only reconstruct the illumination patterns in areas where the object is present, as we only observe the light emitted from the modulated object. If there is no object to interact with the illumination pattern, then no signal is collected in those areas as the illumination light is filtered out. This is a basic principle of fluorescence microscopy.

In Supplementary Figure 4, ground-truth and UBSIM reconstructed images are shown for two examples. The first column displays the illumination patterns. A visual assessment of the ground-truth and UBSIM images shows that some of the intensity maxima in the images seem to be colocated, indicating similarity. However, some regions do not match. Given that we can only reconstruct the areas of the illumination patterns where the object is located, we then display the ground-truth and reconstructed objects and binary masks which indicate where the object images are above a small threshold value. These masks are then multiplied with the illumination patterns to indicate the regions where the illumination patterns should be accurately reconstructed. A comparison of these masked images shows that the local intensity maxima and minima match closely, indicating that UBSIM is able to reconstruct the underlying speckle-patterns.

S10: Evaluation of iteration block number on performance

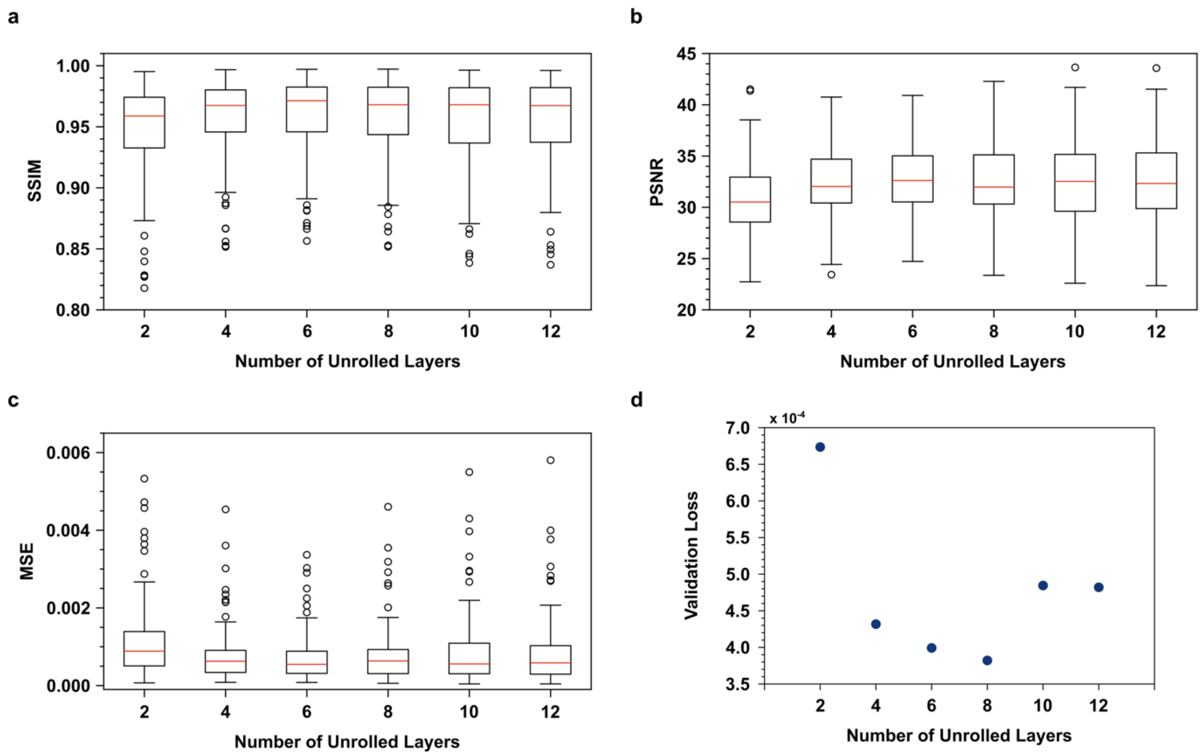

Supplementary Figure 5: Evaluation of iteration block number on performance. Various performance metrics versus models with varying iteration block number. All models are trained for 200 epochs with 20 sub-frames. **a,** SSIM **b,** PSNR **c,** MSE **d,** final validation loss during training. Boxplots are generated using n = 100 independent samples where each sample is a simulated test image not included in the training or validation datasets. The center line indicates the median, the box edges denote the first and third quartiles, and the whiskers extend to the maximum and minimum datapoints within 1.5x the interquartile range. Outlier points are plotted beyond the whiskers.

We explored the effect the number of iteration blocks (unrolled layers) has on model performance through various metrics. We found that the network appears to have optimal performance in the range of 4-8 unrolled layers. Performance metrics improve as the number of layers increases initially, but then start to deteriorate at larger layer numbers. This drop in performance could be due to vanishing gradients or numerical instability. Additionally, increasing the number of layers in an unrolled network can require prohibitively large GPU memory to compute backpropagation. We selected 6 layers for the network used in this paper.

S11. Imaging system experimental setup

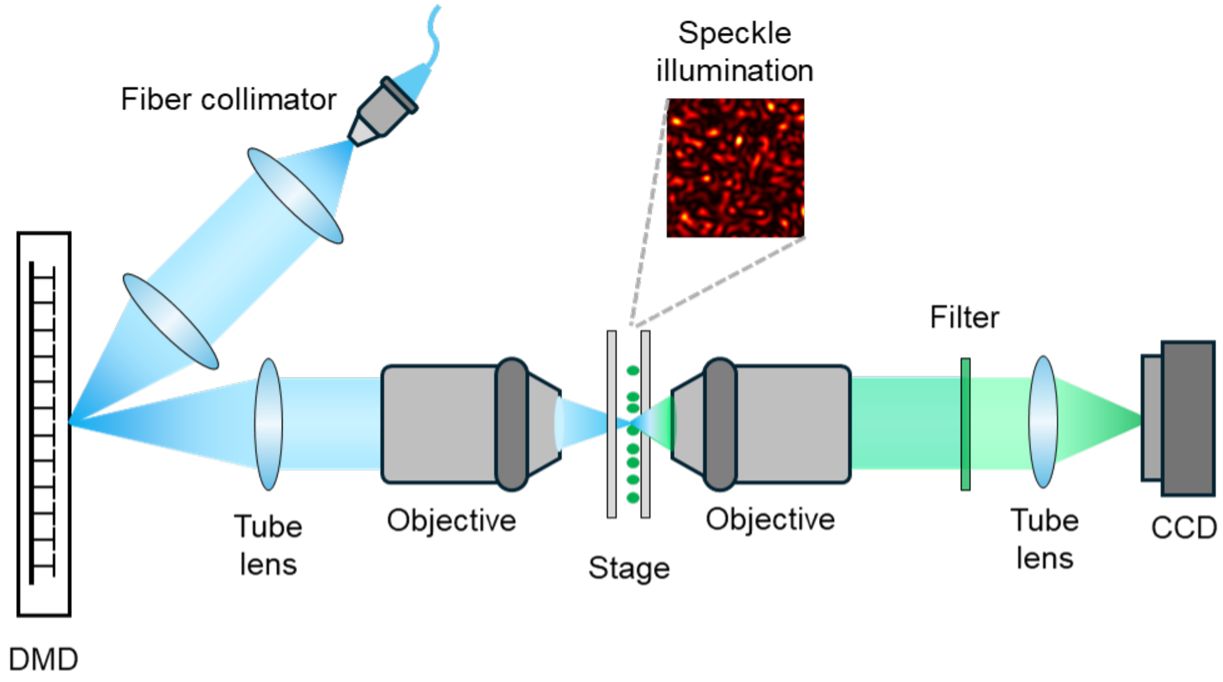

Supplementary Figure 6: Optical path for imaging system. Excitation laser is coupled into a multimode fiber. The light is then outcoupled from a fiber collimator and projected onto a digital micromirror device (DMD) to create illumination patterns. Random binary patterns are displayed on the DMD to create spatially varying speckle-like illumination. The illumination patterns are projected onto the sample plane using a tube lens and objective lens. Biological samples are contained between two glass slides in a buffer solution for live-cell imaging. The sample is imaged from the other side by a second objective and the lower wavelength excitation light is filtered out. A tube lens forms the image of the sample on a CCD. The DMD and CCD are synchronized such that a constant pattern is displayed during each exposure time.

Supplementary References:

1. Mudry, E. et al. Structured illumination microscopy using unknown speckle patterns. *Nat. Photonics* **6**, 312–315 (2012)
2. Ayuk, R. et al. Structured illumination fluorescence microscopy with distorted excitations using a filtered blind-SIM algorithm. *Opt. Lett.* **38**, 4723–4726 (2013).
3. Min, J. et al. Fluorescent microscopy beyond diffraction limits using speckle illumination and joint support recovery. *Sci. Rep.* **3**, 2075 (2013).
4. Yeh, L.-H., Tian, L. & Waller, L. Structured illumination microscopy with unknown patterns and a statistical prior. *Biomed. Opt. Express* **8**, 695–711 (2017)
5. Labouesse, S. et al. Joint reconstruction strategy for structured illumination microscopy with unknown illuminations. *IEEE Trans. Image Process.* **26**, 2480–2493 (2017).
6. Idier, J. et al. On the superresolution capacity of imagers using unknown speckle illuminations. *IEEE Trans. Comput. Imaging* **4**, 87–98 (2017).
7. Mangeat, T. et al. Super-resolved live-cell imaging using random illumination microscopy. *Cell Rep. Methods* **1**, 100009 (2021).
8. Xypakis, E. et al. Deep learning for blind structured illumination microscopy. *Sci. Rep.* **12**, 8623 (2022).
9. Guillaume, G. et al. Fast super-resolved reconstructions in fluorescence random illumination microscopy (RIM). *IEEE Trans. Comput. Imaging*, (2024).
10. Goodman, J. W. Some fundamental properties of speckle. *JOSA* **66**, 1145-1150 (1976).
11. Nocedal, J., & Wright, S. *Numerical Optimization*. (Springer, New York, 2006).
12. Nesterov, Y. A method of solving a convex programming problem with convergence rate O(1/k^2). *Sov. Math. Dokl.* **27**, 372–376 (1983).
13. Polak, E. and Ribière, G. Note sur la convergence de méthodes de directions conjuguées. *Rev. Francaise Informat. Recherche Opérationnelle* **3**(16), 35–43 (1969).
14. Ronneberger, O., Fischer, P. & Brox, T. U-net: convolutional networks for biomedical image segmentation. *Med. Image Comput. Comput. Assist. Interv.* **9351**, 234–241 (2015).
15. Qiao, C. & Li, D. BioSR: a biological image dataset for super-resolution microscopy. https://doi.org/10.6084/m9.figshare.13264793.v7 (2020).
