# Supplementary material for: High-speed blind structured illumination microscopy via unsupervised algorithm unrolling
Source: Nat Commun. 2026 Jan 23;17:1967. doi: 10.1038/s41467-026-68693-w (PMC12932644; doi:10.1038/s41467-026-68693-w)
Supplement: Supplementary file 2 — Description of Additional Supplementary Files [file 41467_2026_68693_MOESM2_ESM.pdf]

Description of Additional Supplementary Files

Supplementary Movie 1: A 50 Hz UBSIM reconstructed video of endoplasmic reticulum dynamics in a COS-7 cell transfected with ER-mNeonGreen.

Supplementary Movie 2: A 50 Hz UBSIM reconstructed video of endoplasmic reticulum dynamics in a COS-7 cell transfected with ER-mNeonGreen.

Supplementary Movie 3: A 10 Hz UBSIM reconstructed video of endoplasmic reticulum dynamics in a COS-7 cell transfected with ER-stagRFP.

Supplementary Movie 4: A 10 Hz UBSIM reconstructed video of endoplasmic reticulum dynamics in a COS-7 cell transfected with ER-stagRFP.

Supplementary Movie 5: A 10 Hz UBSIM reconstructed video of endoplasmic reticulum dynamics in a COS-7 cell transfected with ER-stagRFP.
